# A need-based approach to self-management education for adults with co-morbid diabetes and chronic kidney disease

**DOI:** 10.1186/s12882-019-1296-z

**Published:** 2019-04-02

**Authors:** Edward Zimbudzi, Clement Lo, Peter G. Kerr, Sophia Zoungas

**Affiliations:** 1 Department of Nephrology, Monash Health, Melbourne, Australia; 2 School of Public Health and Preventive Medicine, Monash University, Alfred Centre, 99 Commercial Road, Melbourne, 3004 Australia; 3 The George Institute for Global Health, University of Sydney, Sydney, Australia; 4 Diabetes and Vascular Medicine Unit, Monash Health, Melbourne, Australia

**Keywords:** Diabetes, Chronic kidney disease, self-management education, Patient engagement, Patient-centred care

## Abstract

**Background:**

Self-management education needs have not been assessed in patients with complex co-morbid conditions such as diabetes and chronic kidney disease (CKD). The objectives of this study were to 1) determine the self-management education needs for patients with co-morbid diabetes and CKD and 2) co-develop an educational resource meeting the self-management education needs of patients with co-morbid diabetes and CKD.

**Methods:**

Patients with co-morbid diabetes and CKD attending a co-designed, patient-centred outpatient diabetes and kidney clinic at a tertiary metropolitan hospital were recruited for semi-structured interviews. Maximal variation sampling was used, ensuring adequate representation of different gender, age, diabetes duration and stage of CKD. Data were thematically analysed using grounded theory.

**Results:**

Forty-two patients participated. Most were male (67%) and the mean age was 64.8 (11.1) years. The majority of patients preferred an educational resource in the form of a Digital Versatile Disc (DVD) and they thought that current education could be improved. In particular patients wanted further education on 1) management of diabetes and kidney disease (including nutrition and lifestyle, and prevention of the progression of kidney disease) and 2) complications of comorbid diabetes and kidney disease.

**Conclusion:**

Patients with co-morbid diabetes and kidney disease have education gaps on the management of, and complications of diabetes and kidney disease. Interventions aimed at improving patient education need to be delivered through education resources co-developed by patients and health staff. A targeted education resource in the form of a DVD, addressing these needs, may potentially close these gaps.

**Electronic supplementary material:**

The online version of this article (10.1186/s12882-019-1296-z) contains supplementary material, which is available to authorized users.

## Background

The terms ‘self-management education’, ‘self-management support’ and ‘patient education’ are often used interchangeably especially when describing the management of patients with diabetes. Diabetes self-management education (DSME) is designed to help patients develop skills and techniques to enhance diabetes self-care [1–3] leading to improved clinical and self-reported outcomes such as health related quality of life [4]. Diabetes self-management support (DSMS) refers to the support that is required for implementing and sustaining coping skills and behaviours needed to self-manage [2, 3]. In contrast, patient education primarily involves increasing a patient’s knowledge about a disease in order to change behaviour [5]. Self-management education underpinned by self-management support and patient education are paramount for acquisition of problem-solving skills that empower patients to self-care [6, 7].

Assessment of self-management education needs among patients with chronic diseases such as diabetes [8] and chronic kidney disease (CKD) [9] has indicated a wide variation between the information made available to patients and their specific knowledge needs. For example, studies in patients with CKD [10, 11] have highlighted gaps in awareness of the disease while another study reported poor self-management education levels among patients with diabetes [8].

Patient involvement in the development of self-management education resources may ensure content is relevant, understandable and actionable. Indeed, previous studies among patients with diabetes [12, 13] highlight the importance of seeking patients’ perspectives on what they value about an education intervention and the requirement for a needs assessment before the development of self-management education resources. One study [14] suggested the importance of considering patients’ different knowledge ‘starting points’ and the origins of their knowledge deficits as these are likely to inform how patients engage with, and comprehend education.

Although patient self-management education needs have been assessed for single diseases such as diabetes [15] and CKD [16], they have not been assessed for complex co-morbid conditions such as diabetes and CKD. This is despite the fact that self-management may be particularly important for the outcomes of this group of patients [17]. People with complex co-morbid diseases may have competing self-management strategies and challenges [18], which put them at risk of negating the management of other conditions especially later diagnoses. This can be explained by the concept of “dual task theory” where individuals are likely to perform self-care tasks for conditions in which they have an emotional investment at the expense of others [19]. For example, patients with diabetes and CKD may pay particular attention to the management of diabetes at the expense of kidney disease. In this regard, robust, pragmatic and patient-centred self-management educational tools for patients with co-morbid diabetes and CKD are required.

The overarching objectives of the present study were to 1) qualitatively determine the self-management education needs for patients with diabetes and CKD and 2) co-develop an educational resource meeting the self-management education needs of patients with co-morbid diabetes and CKD.

## Methods

### Design and setting

**Fig. 1:**
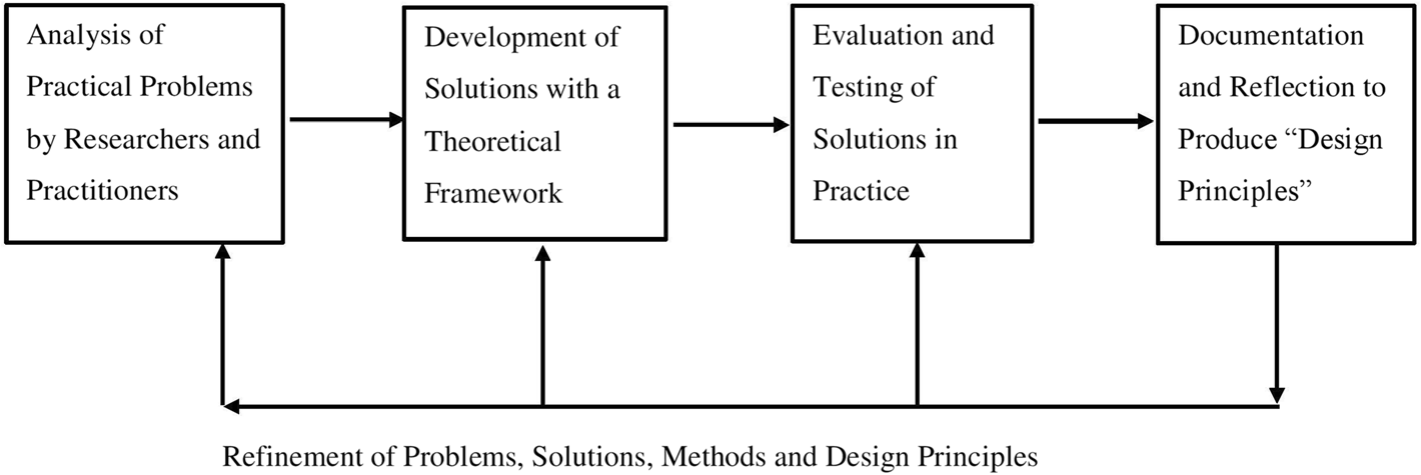
Development research (Design-Based Research)-adapted from Reeves (2000)

We utilised a design-based research (DBR) framework [20] to develop an educational resource in the form of a Digital Versatile Disc (DVD) for patients with co-morbid diabetes and CKD (Fig. 1). The DBR approach allowed researchers, practitioners, patient advocate groups and patients to be more directly engaged in the conduct of the research as well as providing a platform for the cyclic nature that enabled the continual collaboration between all groups of people involved [20]. Patients involved in the study were attending the Diabetes Kidney Service (DKS), an outpatient diabetes and kidney clinic of a tertiary referral hospital. Recruitment took place over a three months period from June to August 2017.

### Patients

Eligible patients were at least 18 years of age, had a diagnosis of diabetes (either type 1 or type 2) and CKD stages 3 to 5 (eGFR< 60 mL/min/1.73 m^2^) including dialysis. The CKD-EPI formula [21] was used to estimate eGFR. The diagnosis of diabetes followed the World Health Organisation definition [22] and was recorded from patients’ prior inpatient or outpatient contacts. Patients were excluded from the study if they could not speak fluently in English and had cognitive impairment. Patients meeting the inclusion criteria were invited to participate when they presented for their routine diabetes and kidney clinic appointment. We used maximal variation sampling to ensure adequate representation by gender, age, diabetes duration and stage of CKD. The interviewer (EZ) was a registered nurse and PhD student who did not provide clinical care to the patients in the clinic setting. The interviewer had received formal training in qualitative research methods.

### The diabetes kidney service

The Diabetes Kidney Service [23], launched in 2015 is a co-designed model of care, tailored to the needs of patients, their care givers, and health-professionals. It is staffed by an interdisciplinary team including endocrinologists, nephrologists, nurse practitioners and a dietitian. Patients are referred to the service from general practice, following hospital admissions and from existing diabetes and nephrology clinics. Eligibility for referral include an eGFR< 60 ml/min/1.73 m^2^ and diabetes. Patients referred to this integrated clinic do not need to be seen in individual endocrine and nephrology clinics unless they are discharged back to these services at their request. Apart from providing clinical care, the interdisciplinary clinic uses a person-centred approach for self-management education for patients and their families. Patient education is delivered verbally or through standard pamphlets and brochures. Interventions embedded within the Diabetes Kidney Service are expected to improve patient outcomes such as slowing CKD progression, better glycemic control and increased patient satisfaction from attending one clinic instead of multiple clinics.

### Semi-structured interviews

Semi-structured interviews (of 15 to 20 min duration) were conducted amongst patients to determine the information required by patients to facilitate self-management of co-morbid diabetes and CKD. One question was close-ended, and three questions were open-ended (Additional file 1). The closed-ended question assessed patients’ preferences of watching a DVD if it was available as a mode of delivering self-management education. Open-ended questions assessed overall the self-management education needs for patients with co-morbid diabetes and CKD and prompted them to highlight questions they would like a diabetes/kidney disease expert to address. Semi-structured interviews were conducted until thematic saturation was reached. Verbatim reports of the conversations were written during the interviews and transcripts were de-identified.

Transcripts underwent thematic analysis independently by two researchers (CL and EZ), informed by grounded theory [24]. Themes in the data were identified using an inductive approach. The resultant themes were reviewed by a multidisciplinary team (endocrinologists, nephrologists, diabetes and renal nurse practitioners and a dietician) and key stakeholders. The key stakeholders included a Clinical Director and Project Officer for Kidney Health Australia and consumer representative for Diabetes Australia.

### Script production

Using the identified educational needs two authors (EZ and CL) drafted the script for the DVD which was then reviewed by the other authors (including Endocrinologists and Nephrologists) and the consumer advocacy groups (Diabetes Australia and Kidney Health Australia) in an iterative process until all were happy with the script. The DVD script was written at 6th grade level to allow comprehension by patients at all levels of health literacy (Additional file 2).

### Ethical considerations

Monash University and Monash Health Human Research Ethics Committees approved the study. Patient data was de-identified and treated confidentially.

## Results

Forty-two patients participated. Most were male (67%) and the mean age was 64.8 (11.1) years. Patients were born in 14 different countries with the majority having been born in Australia (41%). Chronic kidney disease stages 3a–5 including those on dialysis were represented as follows: 3a (24%), 3b (36%), 4 (21%) and 5 (19%). Demographic and clinical characteristics are presented in Table 1.

**Table 1:** Characteristics of interview patients

Characteristic	*N* = 42
Age, mean (SD)	64.8 (11.1)
Male, %	28 (66.7)
Country of birth, *N* (%)	
Australia	17 (40.5)
Cambodia	1 (2.4)
England	2 (4.8)
Germany	2 (4.8)
India	3 (7.1)
Italy	1 (2.4)
Mauritius	4 (9.5)
Malaysia	1 (2.4)
New Zealand	2 (4.8)
Samoa	3 (7.1)
Serbia	1 (2.4)
Sri Lanka	3 (7.1)
Turkey	1 (2.4)
Vietnam	1 (2.4)
Type of diabetes, N (%)	
Type 1	2 (4.8)
Type 2	40 (95.2)
Diabetes duration (years), mean (SD)	18.0 (9.2)
Stage of kidney disease	
3a	10 (23.8)
3b	15 (35.7)
4	9 (21.4)
5 (not on dialysis)	1 (2.4)
5 (on hemodialysis)	7 (16.7)

*N* = number of patients, *SD* = standard deviation

The majority of participants preferred an educational resource in the form of a DVD if it was made available at clinic while a few wanted to watch general television while waiting to be reviewed.

“Yes, I would benefit from watching something educational” (Patient 6).

“I prefer watching the TV. I can get education from the internet” (Patient 15).

The interview data produced 20 codes, which resulted in three main themes. The themes were varying patient satisfaction with current resources, limited knowledge on management of diabetes and kidney disease and inadequate knowledge on complications of diabetes and kidney disease (Table 2).

**Table 2:** Categories and themes derived from the interview data

Categories	Themes
Wanting to know more about new educational materials	1. Varying patient satisfaction with current resources
Current educational materials inadequate	
General knowledge about diabetes and kidney disease management	2. Limited knowledge on management of diabetes and kidney disease i. General knowledge ii. Nutrition and lifestyle iii. Prevention of the progression of kidney disease
Medications involved	
Role of exercising, fitness and healthy lifestyle	
Diabetes and kidney disease diet	
Complications of diabetes and kidney disease	3. Inadequate knowledge on complications of diabetes and kidney disease
Connection between diabetes and kidney disease	
How to slow down kidney damage	
How diabetes causes kidney disease	

### Varying patient satisfaction with current resources

Some patients were aware of the education materials currently available but had no confidence in them and thought the materials were too prescriptive. Patients also reported that they could benefit from new self-management education resources.

“Current education is prescriptive; must do this or else … ” (Patient 17).

“Most pamphlets are sugar-coated. How can I know the truth?” (Patient 16).

“Want to know more about new educational materials” (Patient 10).

Other patients did not appear bothered by their limited understanding of diabetes and kidney disease. They acknowledged their inadequate knowledge on diabetes and kidney disease and were happy with the current self-management education.

“I don’t know; I listen to what they tell me. I don’t have much trouble with my kidneys. Generally OK” (Patient 22).

On the other hand, some patients, especially those with a longer diabetes duration, expressed satisfaction with current education resources provided by their specialists and that they could access further education from the internet. As such, they did not feel that they could benefit from other forms of education.

“It is going to be repeating what I already know” (Patient 13).

“I see doctors often and do not believe I require further education” (Patient 22).

“I can get the education I want from the internet” (Patient 15).

### Limited knowledge on management of diabetes and kidney disease

#### General knowledge

A number of patients demonstrated limited general knowledge on the management of diabetes and kidney disease. This was especially evident about the treatment of diabetes and kidney disease where several patients thought that these conditions could be ‘cured’.

“I would like to know whether there is a cure for my diabetes and kidneys” (Patient 27).

Other patients needed more education on how to take their current medications as well as interpreting their blood glucose readings.

“How do I titrate my insulin?” (Patient 39).

“I want to know how to interpret my blood sugar readings” (Patient 40).

#### Nutrition and lifestyle

Nutrition and lifestyle were mentioned by most patients, including those with longer diabetes duration. They understood that adhering to specific diets was important to successfully self-manage diabetes and kidney disease. They also emphasised the importance of being educated about the necessary diets and expressed particular knowledge gaps regarding these diets.

“I want to know more about diet, fluids and how much sugar to eat” (Patient 20).

“I need education about the diet required to manage kidney disease” (Patient 39).

“I need to know about carb counting” (Patient 37).

“…. how can I live with kidney disease; can I do something about my diet and medications to reduce kidney damage” (Patient 3).

Patients were aware of the importance of healthy lifestyle but wanted to know more about the role of exercise in improving quality of life and health. This knowledge gap was evident even in patients with longer diabetes duration and those with end stage kidney disease and in both men and women.

“I want to know more about fitness and health in general” (Patient 6).

#### Prevention of progression of kidney disease

Several patients demonstrated that they were keen to take some action to slow the progression of kidney disease, but they lacked knowledge on the self-management interventions they were supposed to follow. Other patients also highlighted some interventions they could use to slow kidney disease progression, but they lacked confidence; they needed a health professional to validate their opinion.

“What can I do to prevent further deterioration of my kidneys?” (Patient 1).

“What is the condition of my kidneys and does my weight impact on my kidney function?” (Patient 5).

### Inadequate knowledge on complications of diabetes and kidney disease

A number of patients reported the need for more education regarding prevention of complications associated with co-morbid diabetes and kidney disease. Others seemed to have some knowledge regarding the complications of diabetes and kidney disease, discussing some of these complications and mentioning that kidney disease was the most common complication of diabetes.

“Which body organs are affected by diabetes?” (Patient 4).

“I want to know about diabetes complications such as foot and eye problems” (Patient 16). 

“I need education on complications of diabetes and renal disease” (Patient 20).

“How do I reduce kidney damage from the sugar?” (Patient 25).

While a majority of patients knew that diabetes causes kidney disease, there was a knowledge gap in terms of the actual pathophysiology. Patients felt that this understanding was important in empowering them to improve diabetes control and reduce kidney damage.

“How does diabetes cause kidney disease”? (Patient 40).

“How is diabetes going to affect my kidneys?” (Patient 21).

“How are kidneys affected by diabetes and how to control it so there is no further damage”? (Patient 25).

## Discussion

In this study, we qualitatively assessed the self-management education needs of patients with co-morbid diabetes and CKD through interviews and co-developed an educational DVD for use by patients with these complex chronic disease conditions. The majority of patients preferred an educational resource in the form of a DVD if it was made available and they thought that current education could be improved. In particular patients wanted further education on 1) management of diabetes and kidney disease (including nutrition and lifestyle, and prevention of the progression of kidney disease) and 2) complications of comorbid diabetes and kidney disease.

Our results highlight that the educational needs of patients with co-morbid diabetes and CKD are not currently being met. Patients had general knowledge deficits about their disease, which may be limiting their engagement in the management of their disease. Possible reasons include that currently available self-management education resources may exist in forms that are too hard to understand [25–27] and/or that patients [28, 29] lack of co-ownership of the education resources. There may also be poor acquisition and retention of self-management education especially in the sub-theme of nutrition and lifestyle as previously reported in patients with diabetes [30–32]. We expected that patients with a longer duration of diabetes would have lessor education needs regarding diet compared to those with recent diagnoses of diabetes. However, we did not notice any difference between these two groups of patients suggesting that repetitive educational interventions are needed along the disease continuum to maintain any gains from the initial intervention [32, 33]. Additionally, patients that develop CKD as a complication of diabetes may already have reduced self-management capabilities for managing another condition that develops from suboptimal management of another, leaving them overwhelmed.

The results confirm that there may be fragmentation of patient education resulting in patients opting to concentrate on self-management for one condition and not the other. For example, there were a number of patients who were not aware that diabetes was the cause of their kidney disease and that treating their diabetes could impact the progression of the disease. Patients with complex conditions have been known to have competing self-management strategies and challenges [18, 34], which put them at risk of managing later diagnoses poorly. Patients with multiple chronic conditions often have to prioritise conditions or reconcile their physicians’ advice. In this regard, the provision of self-management education, which covers both diabetes and CKD ensures that patients understand how inter-related these diseases are. This knowledge can potentially improve their self-management capabilities.

However, the main obstacle is that patients have very limited knowledge on treatment and self-management interventions required to reduce kidney disease progression. While there are several self-management interventions that may reduce kidney disease progression such provision of health information, patient education and telephone-based support [35, 36], the challenge is to incorporate the most pragmatic and effective interventions into patient education resources.

In this study some patients indicated that they preferred accessing education from the internet rather than from formal education resources. This attitude may raise concerns among health professionals as the advent of unapproved education resources on the internet may contribute to misunderstanding or incorrect knowledge about disease management particularly complex comorbid diseases such as diabetes and CKD. Indeed, unapproved resources may lead to dangerous practices that contribute to or accelerate disease progression or other adverse outcomes.

The strength of this study lies in the inclusion of patients, key stakeholders and different health specialists in designing the patient education resource. To our knowledge, the education needs of those with co-morbid diabetes and CKD have not previously been reported. Perspectives from patients of different ethnic groups were also captured thereby increasing the generalisability of the results. Additionally, researcher bias was addressed by giving patients an opportunity to verify their responses during the interview process. A limitation of this study is that interviews were conducted in the clinic where patients were receiving care, and hence this setting may have predisposed participants to give positive responses. To address this, all patients were informed prior to their interviews that participation in the study would not affect their medical care and that responses would be kept confidential.

## Conclusions

Patients with co-morbid diabetes and kidney disease, wanted better-quality self-management resources. Additionally, they wanted to be educated on the management and complications of diabetes and kidney disease. These education needs can be addressed through multidisciplinary team involvement and co-designed education resources such as a DVD.

## Additional files

Additional file 1: Semi-structured interview questions. (DOCX 14 kb)

Additional file 2: Patient education script. (DOCX 25 kb)

## Abbreviations

CKD: Chronic Kidney Disease
DBR: Design-Based Research
DSME: Diabetes Self-Management Education
DSMS: Diabetes Self-Management Support
DVD: Digital Versatile Disc
eGFR: estimated Glomerular Filtration Rate
