# Supplementary material for: A need-based approach to self-management education for adults with co-morbid diabetes and chronic kidney disease
Source: BMC Nephrol. 2019 Apr 2;20:113. doi: 10.1186/s12882-019-1296-z (PMC6444589; doi:10.1186/s12882-019-1296-z)
Supplement: Supplementary file 1 — Semi-structured interview questions. (DOCX 14 kb) [file 12882_2019_1296_MOESM1_ESM.docx]

Additional file 1: Semi-structured interview questions

1. Would you benefit from watching a video on information about diabetes and kidney disease self-management? Yes No

2. What topics would you like to be covered in the video with regards to your

a. diabetes

b. kidney disease?

3. If you were to ask a diabetes expert one question, what would this question be?

4. If you were to ask a kidney disease expert one question, what would this question be?
