# Supplementary material for: A need-based approach to self-management education for adults with co-morbid diabetes and chronic kidney disease
Source: BMC Nephrol. 2019 Apr 2;20:113. doi: 10.1186/s12882-019-1296-z (PMC6444589; doi:10.1186/s12882-019-1296-z)
Supplement: Supplementary file 2 — Patient education script. (DOCX 25 kb) [file 12882_2019_1296_MOESM2_ESM.docx]

**Patient education script**

| **Slide** | **Audio** |
| --- | --- |
| 1 | Welcome to the Diabetes Kidney Clinic. My name is ... I am one of the nurse practitioners in the clinic and I can help you look after both of your diabetes and kidney disease. |
| 1 | My name is **…** and I am one the diabetes specialists at the clinic. |
| 1 | My name is **…** and I am one of the kidney specialists at the clinic. |
| 1 | My name is **…** and I am the dietician at the clinic. |
| 2 | Here at the Diabetes Kidney Clinic, we work with you and your local doctor to take care of your diabetes and kidney disease.  You will see either a diabetes specialist or a kidney specialist who will review both your diabetes and kidney disease.  Sometimes you may see both specialists depending on your situation.  Other people you may see during your time in the clinic are the Diabetes Nurse educator, Nurse practitioners (either diabetes or renal) and a Dietician. |
| 3 | In this clinic, a team of staff provides your care.  Staff may come and go while you are being seen, but we will work together to help you. |
| 4 | Education about Diabetes and Kidney Disease |
| 5 | Before we start, it is important to remember that the information shared during this video presentation is of a general nature ONLY.  The aim of this information is to increase your knowledge about diabetes and kidney disease.  It is not intended to replace the advice given to you by your doctor for your particular situation.  It may raise questions that you may wish to ask your diabetes or kidney doctor during your visit to the clinic. |
| 6 | **Diabetes** |
| 7 | You may already know about diabetes, but generally people with diabetes have high amounts of sugar/glucose in their blood. This is because the body is unable to convert the sugar/glucose from food into energy.  The hormone that controls sugar is insulin.  The insulin system does not work properly when you have diabetes - either insulin is no longer produced or not enough insulin is produced by the body. |
| 8 | But, what is insulin?  Insulin is a hormone produced by the pancreas.  The pancreas is an organ located in the middle of the abdomen, below the stomach.  When carbohydrate or sugar in food is digested from the stomach, it can be absorbed into the blood stream as glucose.  Insulin then allows the glucose to be absorbed by the body’s cells either for storage or for use as energy. |
| 9 | You may already know about the two types of diabetes, which are Type 1, and Type 2. I will explain a little bit more about them. |
| 10, 11 | In Type 1 diabetes, the glucose levels become elevated because the part of the pancreas that makes insulin no longer works.  Due to this, no insulin is produced to allow absorption of glucose from the blood into the body’s cells.  Type 1 diabetes occurs when the body’s defence system attacks the insulin producing cells in the pancreas. When the body defence system attacks itself, it is called an autoimmune condition.  Type 1 diabetes occurs in children or young adults but can be diagnosed at any age.  It is also common among people with a family history of type 1 diabetes and those with other autoimmune conditions such as thyroid or coeliac disease. |
| 12, 13 | On the other hand, in Type 2 diabetes, glucose levels become elevated because the body is unable to effectively use the insulin. This is called insulin resistance. In addition, not enough insulin is produced by the pancreas.  Type 2 diabetes usually occurs in middle-aged to older adults although it has been diagnosed in younger people too.  It is more common in people who have a family history of type 2 diabetes,  are overweight, are older, are of particular ethnic groups for example Indigenous Australian, Asian, Pacific Islander, have an inactive lifestyle,  have an unhealthy diet with a lot of fast food and sugar, or have a history of diabetes during pregnancy also called gestational diabetes. |
| 14 | **Complications of Diabetes** |
| 15 | What sort of complications can diabetes cause? |
| 16 | Diabetes can cause some long-term complications such as damaging blood vessels.  Organs affected due to damaged blood vessels include the heart, brain, kidneys, eyes and nerves. |
| 17 | Regarding complications to the heart and kidneys, if you have one complication, you are at greater risk of having others.  The risk factors for heart and kidney complications for diabetes are often the same and they feed off each other and make each other worse.  But the good news is that the treatments to prevent complications are often quite similar. They include managing your diet, lifestyle (such as doing regular exercise), blood pressure, limiting salt, and maintaining the glucose at accepted levels. |
| 18 | Diabetes can also cause blockage of a blood vessel supplying the brain, which can cause a stroke.  The symptoms vary depending on the part of the brain affected.  Typically, if someone with diabetes suddenly develops arm or leg weakness; numbness on one side; difficulty speaking; or droopiness on one side of their face, then a stroke should be suspected.  A stroke is a medical emergency and one would need to be brought to hospital as soon as possible. |
| 19 | Diabetes can cause nerve damage in the feet, causing tingling, burning, or a feeling similar to ants crawling on the feet.  Diabetic related nerve damage in the feet may also cause foot problems such as foot ulcers, foot deformities or even bone infections of the foot.  Foot ulcers or bone infections of the foot are serious and can result in the person losing part of or the whole foot.  To prevent these complications, you should check your feet daily to make sure your feet (and the skin of your feet) have not been damaged. |
| 20 | Symptoms of eye disease may only occur in the late stages of diabetic eyes disease.  It is therefore important to have your eyes checked soon after diagnosis to allow treatment and to prevent these complications from developing and progressing.  Your doctor will advise you on how often you will need your eyes to be checked thereafter. |
| 21 | **Kidney Diseases** |
| 22 | You may be aware of how kidneys work. In short, the kidneys are two bean-shaped organs, each about the size of a fist.  They are located just below the rib cage, one on each side of the spine.  Every day, the two kidneys filter about 180 litres of blood to produce 1 to 3 litres of urine.  Urine flows down through narrow tubes called ureters to the bladder where it is stored until you pass urine. |
| 23 | Functions of the kidneys include, filtering your blood, to remove toxins and balance water, regulating your blood pressure, producing a hormone called erythropoietin to help the body produce red blood cells and activating vitamin D. |
| 24 | People with diabetes are at greater risk of developing kidney disease especially if they have high blood sugar, high blood pressure, lifestyle habits such as smoking and drinking a lot of alcohol, heart disease and a family history of kidney failure. |
| 25 | Kidney disease is often silent until very late in kidney failure. Symptoms of kidney disease may include swelling of hands, legs or more generalized puffiness, poor appetite, nausea and vomiting, weakness, drowsiness, itchiness and rash, and muscle twitchiness. |
| 26, 27 | You can keep your kidneys healthy by monitoring blood pressure, taking blood pressure tablets if prescribed and working with your healthcare team to manage your diabetes as best as you can. You should also try to keep a healthy body weight, exercise regularly, and eat a balanced diet avoiding salt, sugar and saturated fat.  If you smoke, stop smoking- you can call QUIT on 13 78 48 and ask for a free Quit Pack.  Try to lose weight if you are overweight and exercise regularly. |
| 28 | How is kidney disease treated?  If you have diabetes and kidney damage, several things can be done to slow kidney damage.  Apart from maintaining a healthy lifestyle, keep your blood sugar within range. Ask your diabetic specialist what your optimal HbA1C is and keep your blood pressure below 140/90.  If you have diabetes, you can also manage your blood pressure and slow kidney damage by taking medications called angiotensin-converting enzyme inhibitors (ACE) and angiotensin receptor blockers (ARBs). |
| 29 | Your kidneys may eventually fail requiring renal replacement therapy. The 4 treatment options, which are available, are haemodialysis, peritoneal dialysis, transplantation and conservative management.  You may be invited to attend an education session to learn more about these options. |
| 30 | **Diet** |
| 31, 32 | The best diet for patients with diabetes is one that provides adequate nutrition and aims to prevent long term complications while assisting in keeping your blood sugar levels within the target range. |
| 33 | Different types of foods will affect your blood sugar levels.  The amount of food eaten will also affect your blood sugar levels.  There is no specific diet for all people with diabetes.  The diet you may be recommended may be very different to the person sitting next to you! |
| 34 | A few things need to be considered to determine the most appropriate diet for you if you have diabetes. These include the type of diabetes you have Type 1 or Type 2, your age, body weight, medications including glucose lowering medications (including insulin), your activity and whether you are well or unwell or have other illness or disease.  Your food budget and cooking habits are also important considerations. |
| 35 | If you have kidney disease, your stage of kidney disease may mean that your recommended dietary intake may be very different to that of the person sitting next to you.  There is no specific diet for all people with kidney disease. |
| 36 | For people with kidney disease, a few things have to be considered about their diet. These include your weight (including any weight loss or weight gain), your stage of kidney disease, if you are planning to have dialysis and what type, your blood test results and your appetite. |
| 37 | If you have any questions about diet, a dietician is available in the clinic to review your intake, make some recommendations, assist you with getting the right nutrition for your body and assist you with food choices to help you with your diabetes and kidney disease. |
| 38 | Thank you and remember to ask your doctor if you have any questions. |
